# Cav‐1 regulates the bile salt export pump on the canalicular membrane of hepatocytes by PKCα‐associated signalling under cholesterol stimulation

**DOI:** 10.1111/jcmm.18110

**Published:** 2024-01-01

**Authors:** Liwei Pang, Meiying Cui, Shuodong Wu, Jing Kong

**Affiliations:** ^1^ Department of General Surgery Shengjing Hospital of China Medical University Shenyang Liaoning China; ^2^ Department of Anesthesiology, Shengjing Hospital China Medical University Shenyang China

**Keywords:** BSEP, Cav‐1, cell membrane cholesterol, cholesterol cholelithiasis, Hax‐1

## Abstract

**Background and Aims:**

The secretion of bile salts transported by the bile salt export pump (BSEP) is the primary driving force for the generation of bile flow; thus, it is closely related to the formation of cholesterol stones. Caveolin‐1 (Cav‐1), an essential player in cell signalling and endocytosis, is known to co‐localize with cholesterol‐rich membrane domains. This study illustrates the role of Cav‐1 and BSEP in cholesterol stone formation.

**Methods:**

Adult male C57BL/6 mice were used as an animal model. HepG2 cells were cultured under different cholesterol concentrations and BSEP, Cav‐1, p‐PKCα and Hax‐1 expression levels were determined via Western blotting. Expression levels of *BSEP* and *Cav‐1* mRNA were detected using real‐time PCR. Immunofluorescence and immunoprecipitation assays were performed to study BSEP and Hax‐1 distribution. Finally, an ATPase activity assay was performed to detect BSEP transport activity under different cholesterol concentrations in cells.

**Results:**

Under low‐concentration stimulation with cholesterol, Cav‐1 and BSEP protein and mRNA expression levels significantly increased, PKCα phosphorylation significantly decreased, BSEP binding capacity to Hax‐1 weakened, and BSEP function increased. Under high‐concentration stimulation with cholesterol, Cav‐1 and BSEP protein and mRNA expression levels decreased, PKCα phosphorylation increased, BSEP binding capacity to Hax‐1 rose, and BSEP function decreased.

**Conclusion:**

Cav‐1 regulates the bile salt export pump on the canalicular membrane of hepatocytes via PKCα‐associated signalling under cholesterol stimulation.

## 1 INTRODUCTION

Gallstone disease is one of the most common disorders treated by general surgery. Primary pathogenesis of gallstone disease includes genetic susceptibility, abnormal lipid metabolism and abnormal gallbladder function. In recent years, an increasing number of studies have reported abnormal cholesterol transport to be a primary cause of cholesterol supersaturation and gallbladder stone formation.^1^, ^2^ The bile salt export pump (BSEP), a member of the ATP‐binding cassette transporter family, plays an important role in cholesterol‐gallstone formation.^3^, ^4^ Caveolae are small, bulb‐shaped plasma membrane invaginations containing membrane‐anchored scaffolding proteins that perform essential functions in endocytosis, transcytosis, maintenance of membrane lipid composition and signal transduction.^5^, ^6^ One such protein is caveolin‐1 (Cav‐1), a key component involved in intracellular cholesterol balance and lipid transport^7^, ^8^ that is located on the canalicular membrane. Some studies^9^, ^10^ showed that trafficking of BSEP to the canalicular membrane depended on the basal activity of kinases such as the protein kinase Cα (PKCα). In addition, Hax‐1, a 34 kDa polypeptide that interacts with a heterogeneous group of proteins associated with metabolic diseases,^11^ was also involved in BSEP internalization from the apical membrane.^12^, ^13^ However, there are no in vitro studies on the relation between BSEP, Cav‐1, p‐PKCα and Hax‐1. Here, we established a model of gallbladder cholesterol‐stones in mice, stimulated the cells with different cholesterol concentrations, and measured the expression levels of BSEP, Cax‐1, p‐PKCα and Hax‐1 in order to understand the potential relation between them.

## 2 MATERIALS AND METHODS

### 2.1 Animal models and diets

Twenty C57BL/6 mice (8 weeks old, male, SPF level, 20–22 g weight, purchased from Weitong Lihua Experimental Animal Technology Co., Ltd., Beijing, China) were employed in this study. All experimental protocols were approved by the research advisory committee of Shengjing hospital. Animals were fed with standard chow and water ad libitum for 1 week, followed by random division in two groups: a control group (*n* = 10, basic feed) and an experimental group (*n* = 10) which was fed a high fat and high cholesterol diet (HFC: 83.5% basic food, 15% fat, 1% cholesterol and 0.5% bile salt, mixed). During the modelling period, the mice fed and drank freely. Five mice from each group were extracted after 2 and 4 weeks for experimental purposes.

### 2.2 Liver sampling

Mice, after 12 h of fasting were anaesthetised and subjected to blood extraction from the inferior vena cava, before beingculled. Livers were removed, cut into small pieces and washed extensively with cold phosphate‐buffered saline solution. Liver tissues were stored at −80°C until processed.

### 2.3 Cell culture

HepG2 cells were purchased from the Shanghai Cell Bank of the Chinese Academy of Sciences and cultured in MEM/EBSS basic medium (Hyclone Laboratories, Inc., Logan, UT, USA), 10% fetal bovine serum (Biological Industries, Beit HaEmek, Israel), and double antibody (Hyclone Laboratories, Inc.), in an environment with 5% carbon dioxide at 37°C. Cells were seeded at a density of 1 × 10^6^ cells/well in 6‐well plates and cultured for 24 h until 70%–80% confluence was reached. Cells were treated for 48 h with cholesterol solutions at different concentrations to establish our cellular model.

### 2.4 Cholesterol stock solution

A 25 mg/mL of cholesterol stock solution was prepared by accurately weighing 100 mg cholesterol and dissolving it in 4 mL of anhydrous ethanol. The dissolution process was slow and needed to be carried out at 37°C in a water bath. The cholesterol stock solution prepared using this method might precipitate once added to the cell culture medium. To increase its solubility, we added 10% of the total volume of Tween‐80 into the cholesterol stock solution as a co‐solvent. The final concentration of anhydrous ethanol in the subsequent culture medium was 0.1%, and the concentration of Tween‐80 was 0.01%; these concentrations did damage the cells at all.

### 2.5 Cav‐1 overexpression and knockout in vitro

HepG2 cells were transfected with a *Cav‐1* gene overexpression virus and a *Cav‐1* shRNA interference virus, purchased from Jikai Gene Chemical Technology Co., Ltd. (Shanghai), in MEM/EBSS basic medium for 24 h at 37°C. Viral vectors included a GFP‐fluorescent tag and a puromycin resistance gene for measurement of infection efficiency and screening of monoclonal cells. Infection medium was removed, and fresh complete growth medium was added. Expression of the GFP‐tagged protein was observed under an inverted fluorescence microscope at 24 h postinfection to verify the infection efficiency. Subsequently, GFP‐labelled cells were split into 10 cm dishes in a medium containing puromycin; the medium was changed every day until the growth of puromycin‐resistant cells was evident. Individual cells were transferred into 6‐well plates to continue incubation with the puromycin selection medium. Later, they were evaluated for Cav‐1 expression, and this monoclonal line was utilized for all experiments.

### 2.6 Western blot analysis

Proteins were extracted from liver tissues and HepG2 cells using a cell membrane protein and plasma protein extraction kit (Beyotime, P0033), and the following antibodies were used for Western blot (WB) analyses: BSEP polyclonal antibody (PA5‐78690, Invitrogen, Carlsbad, CA, USA); caveolin‐1 antibody (Cell Signaling Technology, Inc., Danvers, MA, USA); anti‐Hax‐1 antibody (Ab137613, Abcam plc., Cambridge, UK); PKCα antibody (Cell Signaling Technology, Inc.); phospho‐PKCα/β II (Thr638/641) antibody (Cell Signaling Technology, Inc.); mouse anti‐GAPDH antibody (Zhongshan Golden Bridge Biotechnology Co., Ltd., Beijing, China); Na,K‐ATPase antibody (Cell Signaling Technology, Inc.); and mouse anti‐β actin antibody (Zhongshan Golden Bridge Biotechnology Co., Ltd.). Protein expression was normalized to β‐actin, GAPDH and Na^+^, K^+^‐ATPase. Densitometry analyses were performed using the ImageJ software.^14^

### 2.7 RNA isolation and real‐time PCR

Total RNA was extracted from cells or tissues using TRIzol reagent (15596018, Invitrogen) according to the manufacturer's instructions. Reverse‐transcription reactions were performed using the PrimeScript™ RT Master Mix (RR036A, Takara Bio Inc., Japan). Primer sequences for BSEP, Cav‐1 and GAPDH are displayed in Table 1.

**TABLE 1:** Primer sequence design.

Gene	Primer	Sequence
mBSEP	Forward primer	ACATCTGTAGGGTTGTTGAGTGA
	Reverse primer	CAAAGAAGCCAACTCGAGCG
mCav‐1	Forward primer	CCCAGGGAAACCTCCTCAGA
	Reverse primer	GCGCGTCATACACTTGCTTC
mGapdh	Forward primer	GGAGAGTGTTTCCTCGTCCC
	Reverse primer	ATGAAGGGGTCGTTGATGGC
hBSEP	Forward primer	AGGACAAGCTGGTCAAGTTTC
	Reverse primer	CCAACTCTAACGCCATCACCT
hCav‐1	Forward primer	CGTAGACTCGGAGGGACATCT
	Reverse primer	TCGTACACTTGCTTCTCGCT
hGapdh	Forward primer	GACAGTCAGCCGCATCTTCT
	Reverse primer	GCGCCCAATACGACCAAATC

### 2.8 Immunoprecipitation assay

To study the interaction between BSEP and Hax‐1, we incubated the cell lysates with anti‐BSEP or anti‐Hax‐1 antibodies and protein A/G‐agarose beads at 4°C with constant rotation overnight; the immunoprecipitated samples were analysed by blotting with anti‐BSEP or anti‐Hax‐1 antibodies.

### 2.9 ATPase activity assay

ATPase activity assays were performed in the presence and absence of sodium orthovanadate (to suppress BSEP) according to the protocol and reagents included in the ATPase Kit (BC0065, Solarbio Science & Technology Co., Ltd., Beijing, China) (see Appendix S1 for specific methods). The employed substrate was taurocholic (TC) acid.

### 2.10 Statistical analysis

Data were expressed as x̄ ± SD and analysed using the Prism v8.0 software (GraphPad Software, San Diego, CA, USA). Independent samples were compared using Student's *t*‐test or ANOVA, and *p ≤* 0.05 was considered statistically significant.

## 3 RESULTS

### 3.1 Mouse BSEP expression is influenced by cholesterol

**FIGURE 1:**
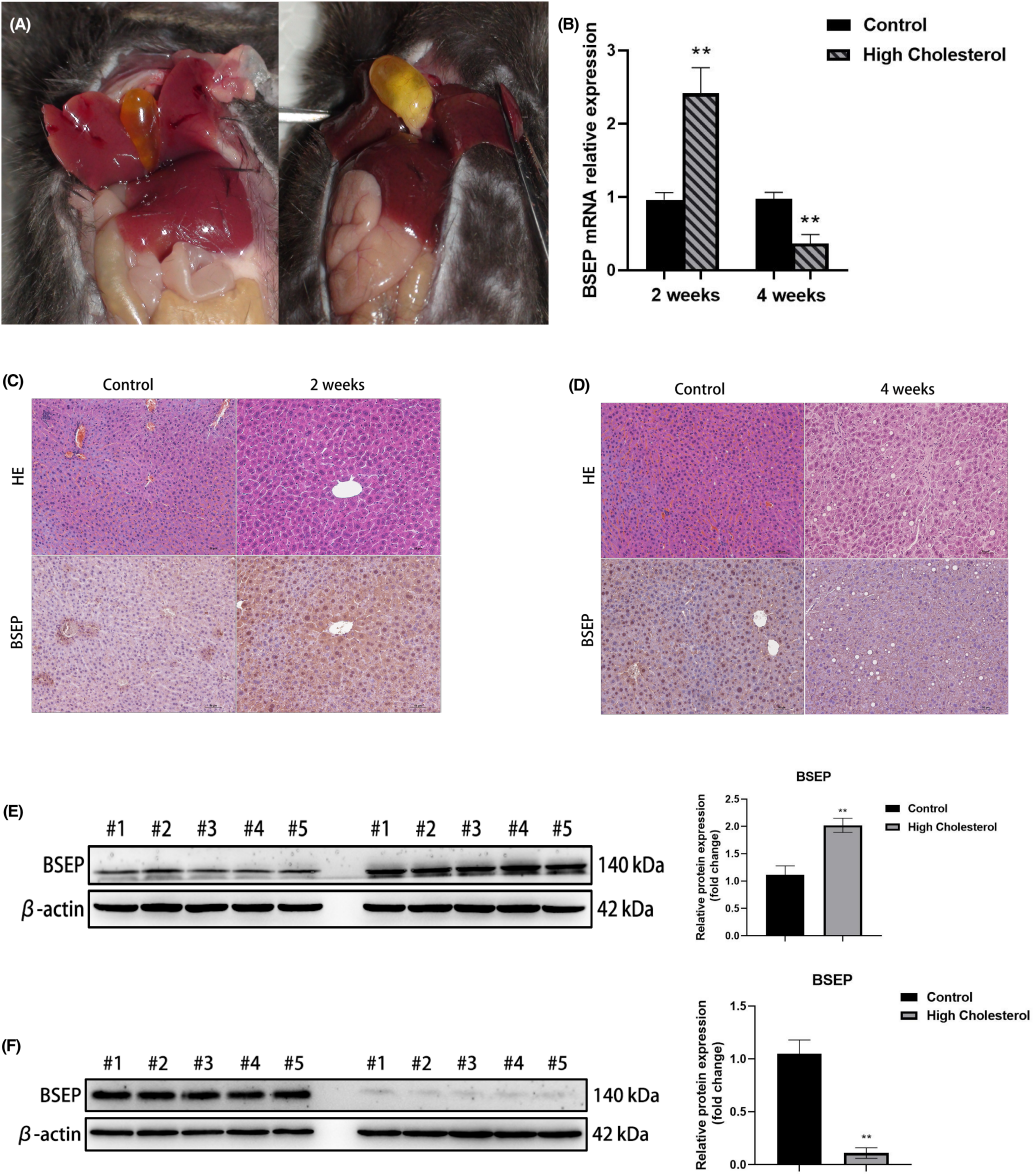
Mouse BSEP expression is influenced by cholesterol. (A) Cholesterol stone in C57BL/6 mice; (B) *BSEP* mRNA expression levels in mice; (C) Immunohistochemistry analysis of BSEP from mouse liver (2 weeks) (200×) shows strong positive expression of BSEP in experimental group; (D) Immunohistochemistry analysis of BSEP from mouse liver (4 weeks) (200×) show a significant decrease in the expression of BSEP in experimental group; (E) Western blot analysis of BSEP from mouse liver (2 weeks) shows increased expression of BSEP; (F) Western blot analysis of BSEP from mouse liver (4 weeks) shows decreased expression of BSEP.

Cholesterol stones were only seen in the gallbladders of C57BL/6 mice that were fed HFC food for 4 weeks (Figure 1A). Immunohistochemistry and WB analysis of mouse hepatocytes showed that BSEP expression increased in the 2‐week experimental group (Figure 1C), while it decreased in the 4‐week experimental group (Figure 1D). Furthermore, *BSEP* mRNA expression was higher in the 2‐week experimental group than in the control group, while *BSEP* mRNA expression in the 4‐week experimental group was lower than in the control group (Figure 1B).

### 3.2 BSEP expression and function in cells is influenced by cholesterol

**FIGURE 2:**
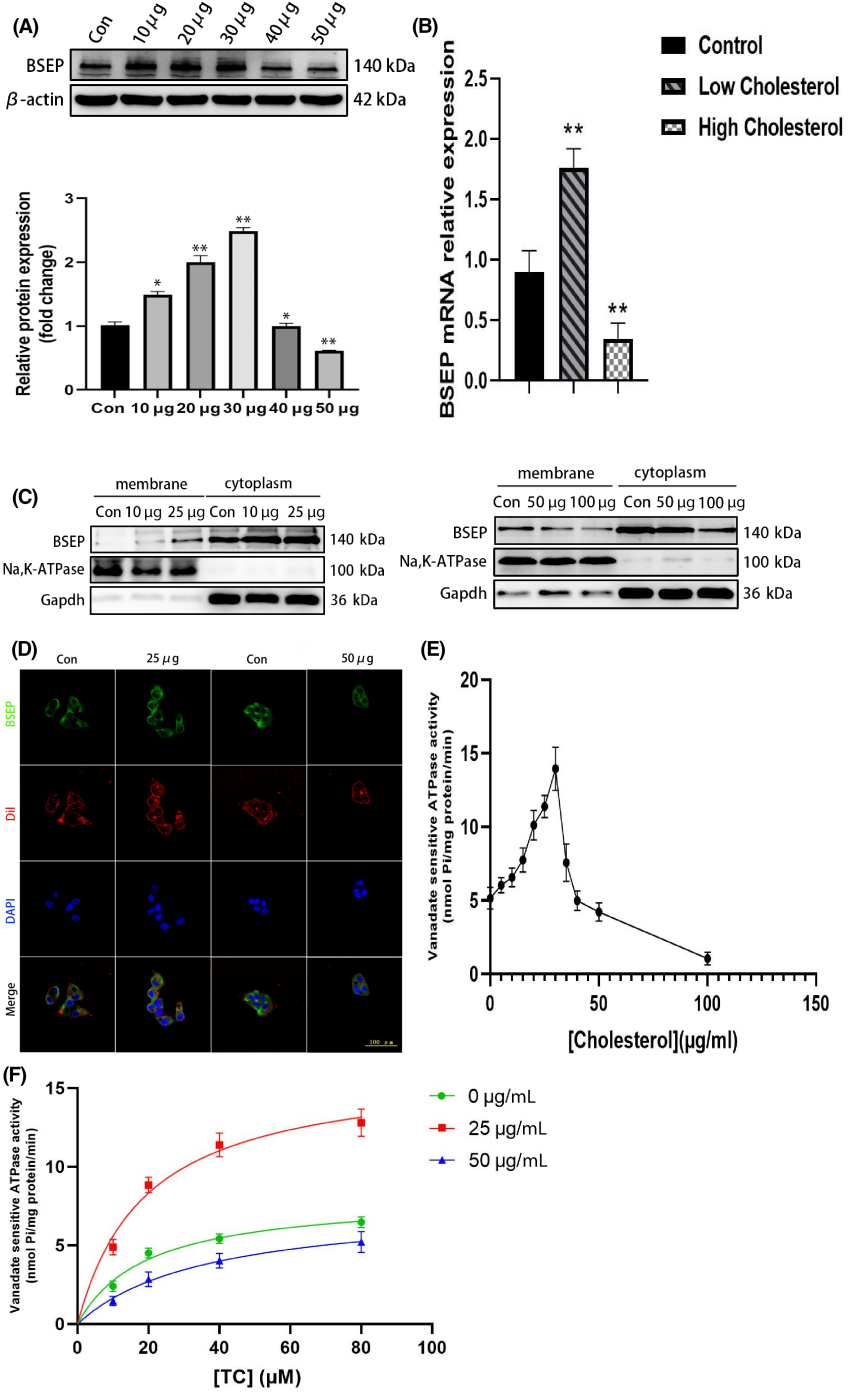
BSEP expression and function in cells are influenced by cholesterol. BSEP protein expression first increased, then decreased as the cholesterol concentration increased. (A) Effects of different cholesterol concentrations on the expression of the BSEP protein in HepG2 cells; (B) *BSEP* mRNA expression levels in HepG2 cells; (C) BSEP protein expression in the cell membrane under different cholesterol concentrations: BSEP protein expression in the cell membrane increased in the low‐cholesterol‐concentration group, but decreased in the high‐ cholesterol‐concentration group; (D) Cell membrane localization of fluorescent BSEP under different cholesterol concentrations: a low‐cholesterol concentration promoted BSEP localization at the cell membrane, while a high‐cholesterol concentration promoted a cytoplasmic localization of BSEP; (E) Effects of cholesterol concentration on BSEP transport activity: BSEP transport activity first increased, then decreased as cholesterol concentration increased; (F) Effect of taurocholic (TC) acid concentration on BSEP transport activity: as the TC substrate concentration increased, BSEP transport activity was higher in the low‐cholesterol‐concentration group than in the control and high‐cholesterol‐concentration groups.

BSEP protein expression first increased, then decreased as the cholesterol concentration increased, and it reached its peak when the cholesterol concentration was approximately 30 μg/mL. However, when the cholesterol concentration was too high (> 30 μg/mL), the cell membrane structure disintegrated and the cell survival rate was also reduced (Figure 2A,B). Therefore, 25 μg/mL was selected as the low‐cholesterol‐concentration experimental group, and 50 μg/mL was selected as the high‐cholesterol‐concentration experimental group in subsequent experiments. *BSEP* mRNA expression was higher in the low‐cholesterol‐concentration group than in the control group, while *BSEP* mRNA expression in the high‐cholesterol‐concentration group decreased. Next, we determined BSEP protein expression in the cell membrane and found that it increased in the low‐cholesterol‐concentration group, but decreased in the high‐cholesterol‐concentration group (Figure 2C). BSEP immunofluorescence analysis also showed that a low‐cholesterol concentration promoted BSEP localization at the cell membrane, while a high‐cholesterol concentration promoted a cytoplasmic localization of BSEP (Figure 2D). Regarding BSEP transport function, at a 40 μM fixed TC concentration BSEP transport activity first increased, then decreased as cholesterol concentration increased (Figure 2E). As the TC substrate concentration increased, BSEP transport activity was higher in the low‐cholesterol‐concentration group than in the control and high‐cholesterol‐concentration groups (Figure 2F).

### 3.3 Mouse Cav‐1 and Hax‐1 expression is influenced by cholesterol

**FIGURE 3:**
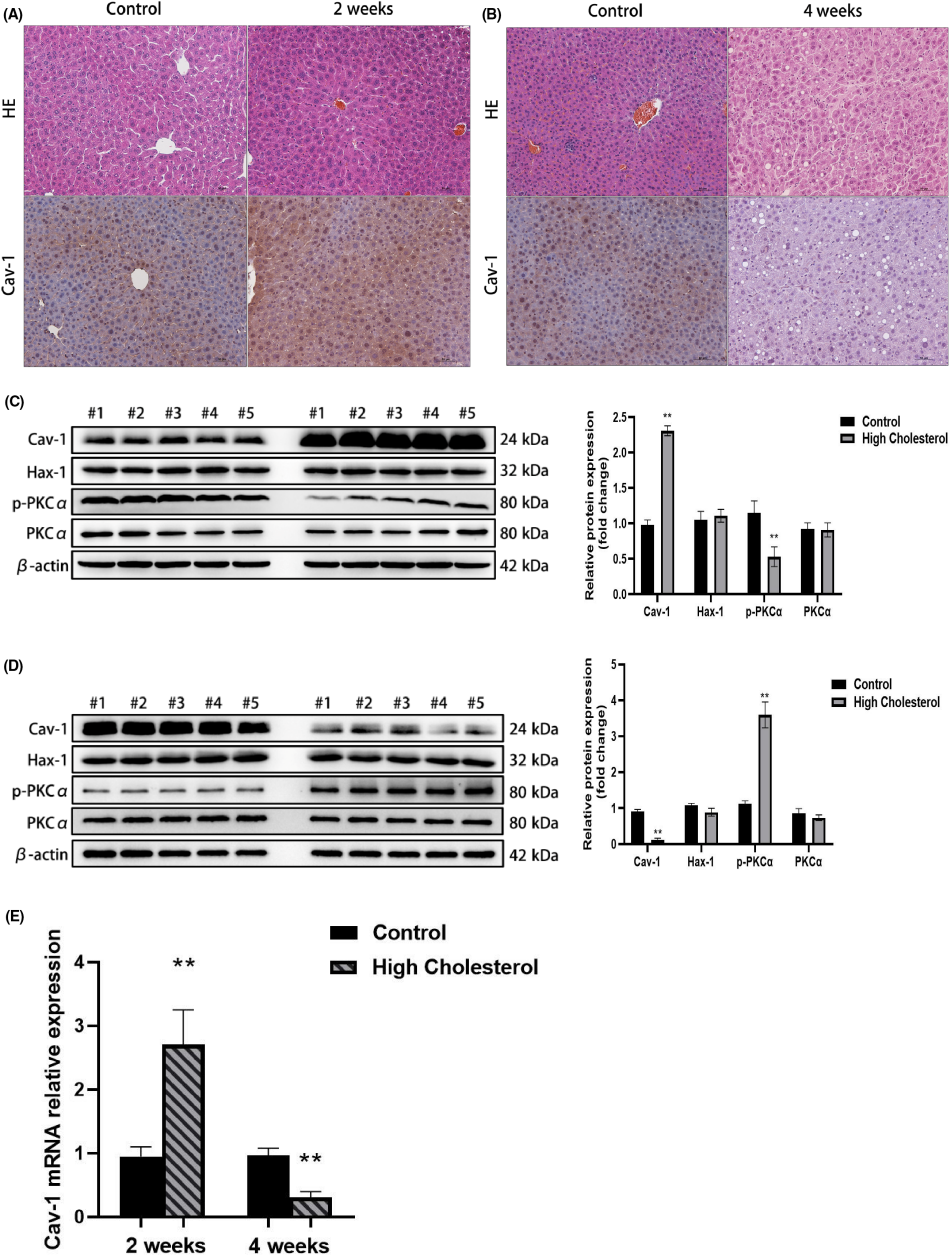
Mouse Cav‐1 expression is influenced by cholesterol. (A) Immunohistochemistry analysis of Cav‐1 from mouse liver (2 weeks) (200×) shows strong positive expression of Cav‐1 in experimental group; (B) Immunohistochemistry analysis of Cav‐1 from mouse liver (4 weeks) (200×) shows significant decrease in expression of Cav‐1 in experimental group; (C) Western blot of Cav‐1, Hax‐1 and p‐PKCα from mouse liver (2 weeks): p‐PKCα in mouse liver was decreased but Hax‐1 expression showed no obvious changes in vivo; (D) Western Blot of Cav‐1, Hax‐1 and p‐PKCα from mouse liver (4 weeks): p‐PKCα in mouse liver was increased but Hax‐1 expression showed no obvious changes in vivo; (E) *Cav‐1* mRNA expression levels in mice: *Cav‐1* mRNA expression was higher in the 2‐week experimental group than in the control group, while Cav‐1 mRNA expression decreased in the 4‐week experimental group compared with the control group.

Immunohistochemistry and WB analysis of mouse hepatocytes showed that Cav‐1 expression was increased in the 2‐week experimental group (Figure 3A), while it decreased in the 4‐week experimental group (Figure 3B). We also found that p‐PKCα in mouse liver (2 weeks) decreased, while p‐PKCα (4 weeks) increased (Figure 3C,D). Furthermore, *Cav‐1* mRNA expression was higher in the 2‐week experimental group than in the control group, while *Cav‐1* mRNA expression decreased in the 4‐week experimental group compared to the control group (Figure 3F). However, Hax‐1 expression showed no obvious changes in vivo.

### 3.4 Cav‐1 and Hax‐1 expression in cells is influenced by cholesterol

**FIGURE 4:**
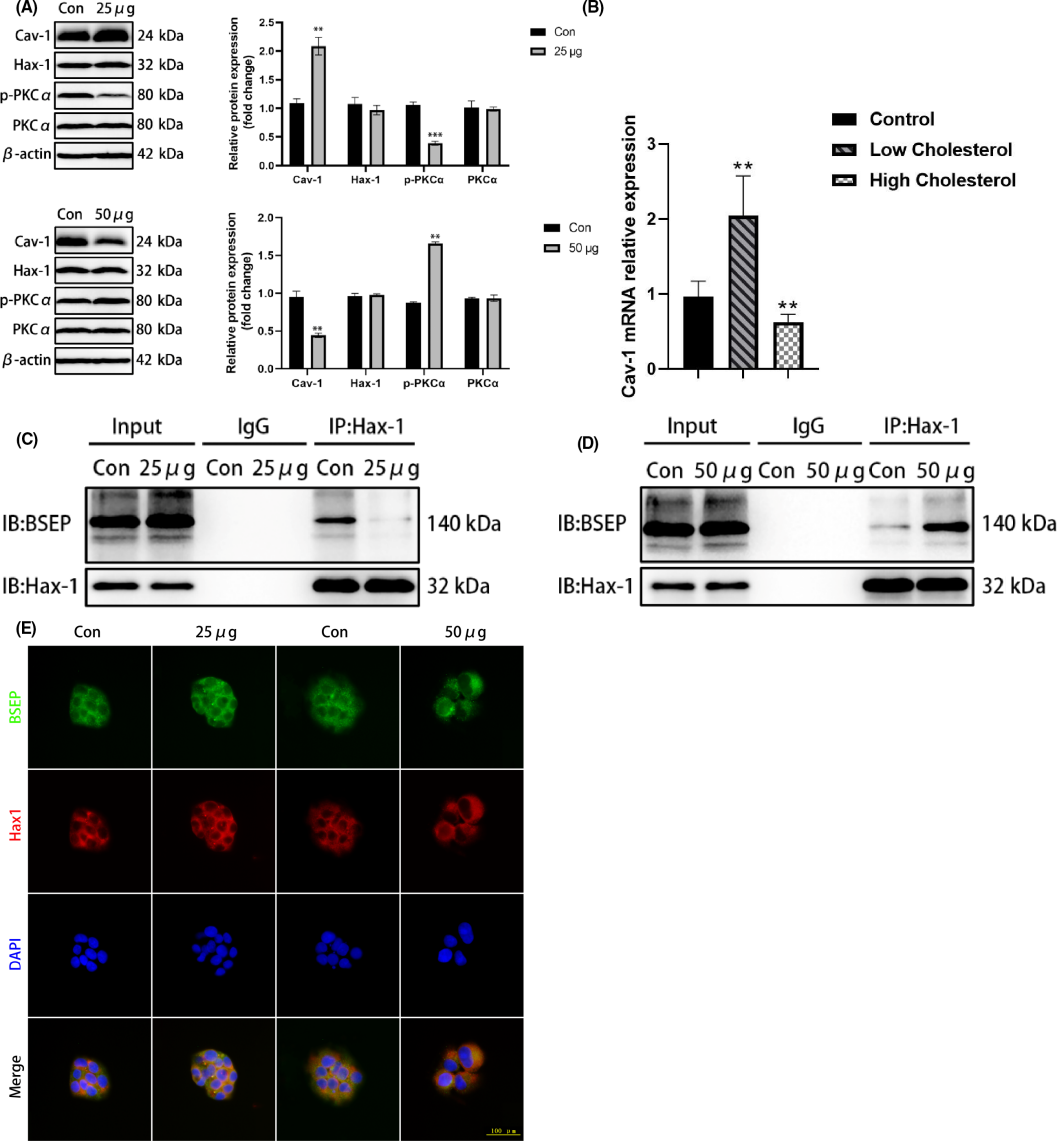
Cav‐1 expression in cells is influenced by cholesterol. (A) Effects of different cholesterol concentrations on Cav‐1 protein expression in HepG2 cells; (B) *Cav‐1* mRNA expression levels in HepG2 cells; (C) BSEP and Hax‐1 co‐immunoprecipitation under low‐cholesterol concentration; (D) BSEP and Hax‐1 co‐immunoprecipitation under high‐cholesterol concentration; (E) BSEP and Hax‐1 double immunofluorescence staining under different cholesterol concentrations.

**FIGURE 5:**
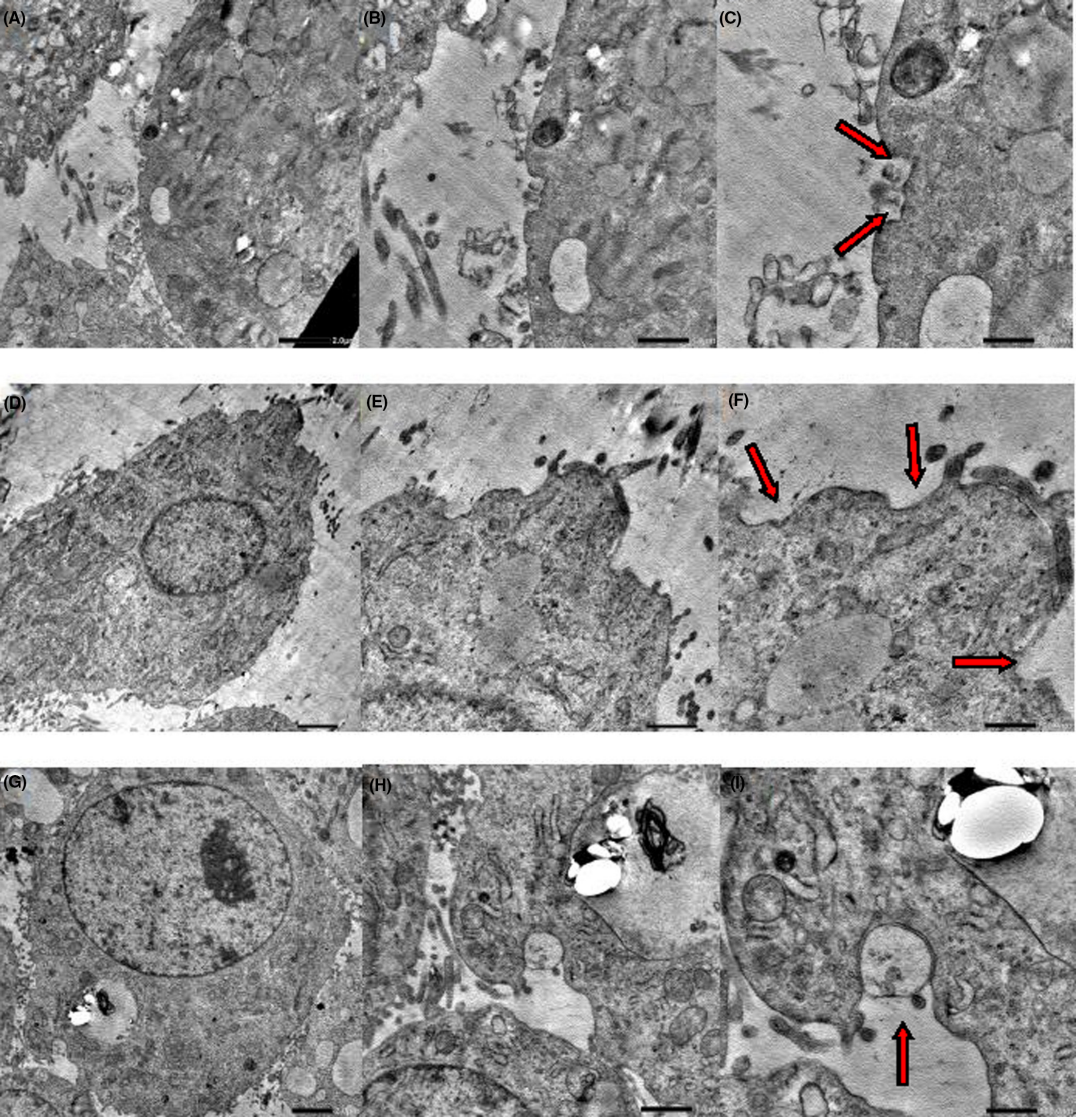
Changes in caveolae of cells under different cholesterol concentrations: low‐cholesterol concentration increased both the number of caveolae and invagination in the cell membrane. Further increase in cholesterol, led to a decrease in cell membrane caveolae, but more invagination was observed. (A) Control group (2500×); (B) Control group (5000×); (C) Control group (10,000×); (D) Low‐cholesterol‐concentration group (2000×); (E) Low‐cholesterol‐concentration group (5000×); (F) Low‐cholesterol‐concentration group (10,000×); (G) High‐cholesterol‐concentration group (2000×); (H) High‐cholesterol‐concentration group (5000×); (I) High‐cholesterol‐concentration group (10,000×).

WB and real‐time PCR analysis showed that Cav‐1 protein and mRNA expression was increased, and PKCα phosphorylation was significantly decreased in HepG2 cells under a low‐cholesterol concentration; under a high‐cholesterol concentration, Cav‐1 expression levels decreased, and PKCα phosphorylation increased. Hax‐1 expression showed no obvious changes in vitro. As cholesterol did not seem to regulate Hax‐1 expression, we considered that it might affect BSEP interaction with Hax‐1 and the localization of BSEP. A co‐immunoprecipitation assay indicated that BSEP ability to bind to Hax‐1 was weakened under a low‐cholesterol concentration, and enhanced under a high‐cholesterol concentration. An immunofluorescence assay further indicated that BSEP and Hax‐1 primarily co‐localized at the cell membrane under a low‐cholesterol concentration, and in the cytoplasm under a high‐cholesterol concentration (Figure 4). Finally, by using a transmission electron microscope, we observed that a low‐cholesterol concentration increased the caveolae and invagination contents in the cell membrane. Further increase in cholesterol led to a decrease in the number of caveolae; however, the invagination content increased (Figure 5).

### 3.5 Effect of Cav‐1 overexpression on BSEP, Hax‐1 and p‐PKCα

**FIGURE 6:**
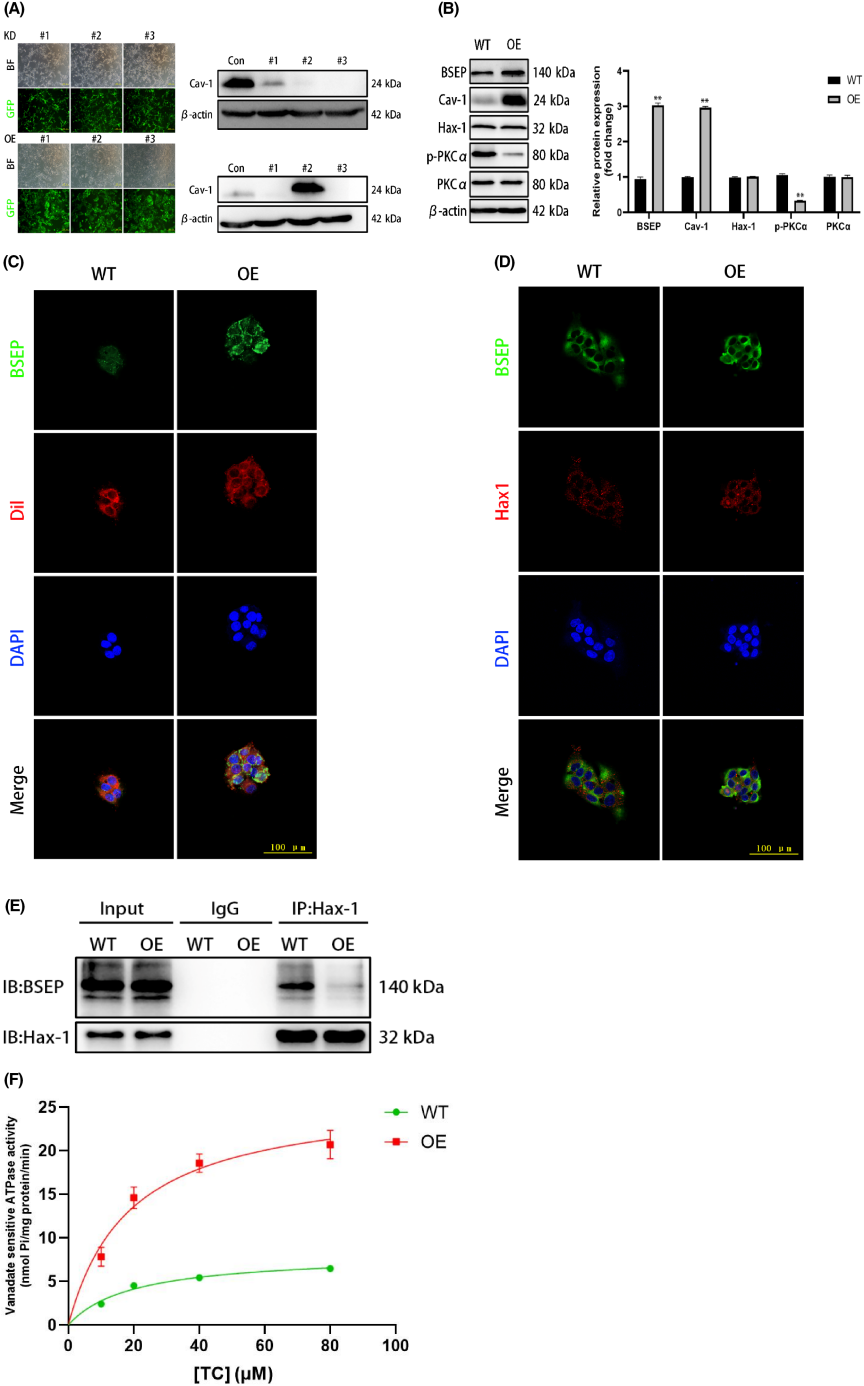
Effect of Cav‐1 overexpression on BSEP, Hax‐1 and p‐PKCα. (A) Establishment and identification of Cav‐1 overexpression and knockdown cell line; (B) Effects of Cav‐1 overexpression on BSEP, Cav‐1, p‐PKCα and Hax‐1 protein expression; (C) Cav‐1 overexpression promoted BSEP cell membrane localization; (D) After Cav‐1 overexpression, BSEP co‐localizes with Hax‐1 in the cell membrane; (E) Cav‐1 overexpression inhibited BSEP binding to Hax‐1; (F) Effects of Cav‐1 on BSEP transport function.

A Cav‐1 overexpression cell line was successfully constructed via lentiviral infection; however, knockdown of Cav‐1 rapidly decreased cell activity and caused cell death. WB analysis showed that because of *Cav‐1* overexpression, BSEP and Cav‐1 protein expressions significantly increased, while PKCα phosphorylation significantly decreased. Once more, Hax‐1 expression showed no obvious changes between the wild‐type and overexpression cell lines. A co‐immunoprecipitation assay indicated that the ability of BSEP to bind to Hax‐1 was weakened, and immunofluorescence analysis further indicated that BSEP and Hax‐1 primarily co‐localized at the cell membrane in the Cav‐1 overexpression cells. Finally, BSEP transport activity in the Cav‐1 overexpression cells was higher than that in the wild‐type cells as concentration of the TC substrate increased (Figure 6).

### 3.6 Rescue experiment

**FIGURE 7:**
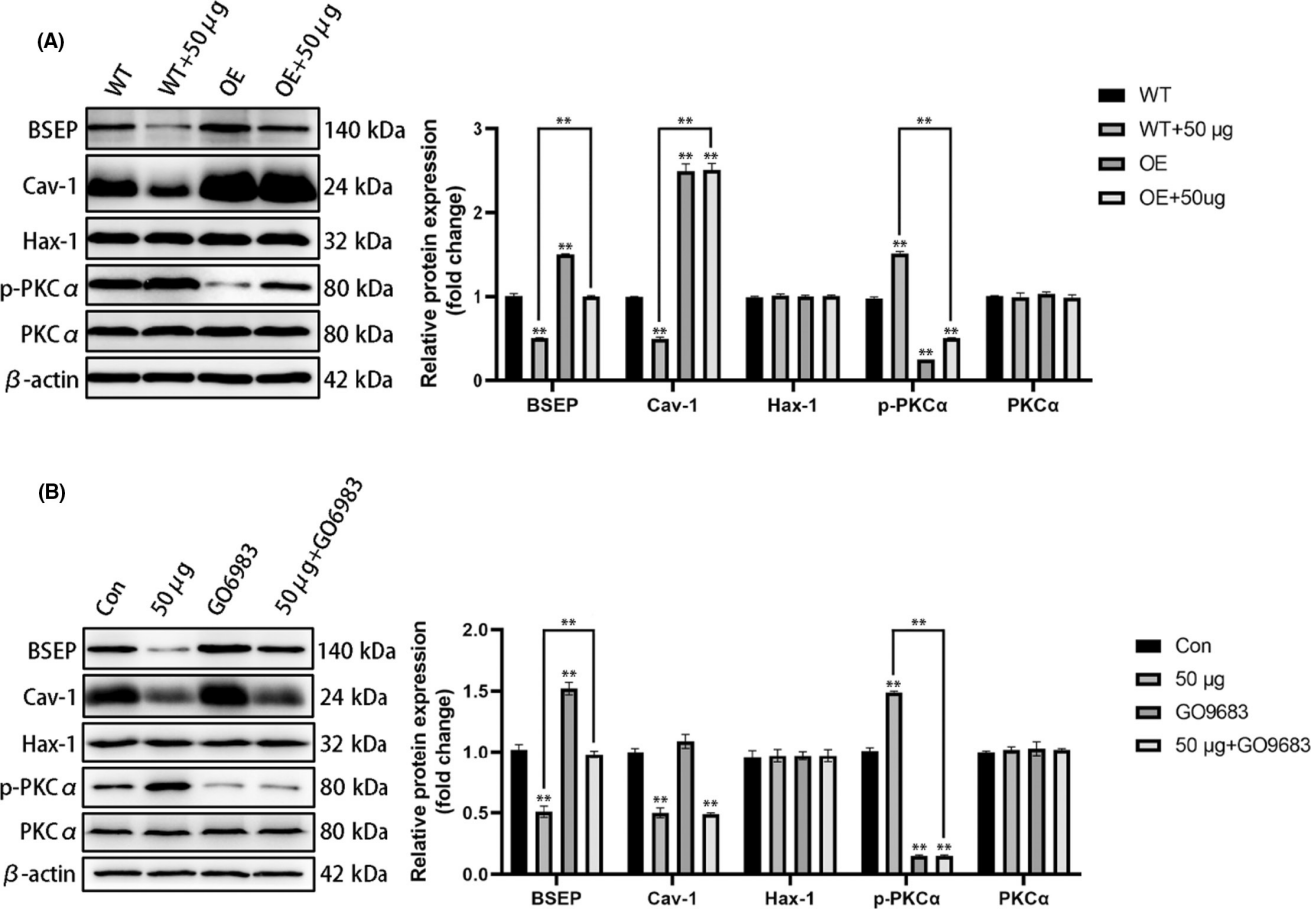
Cholesterol regulated BSEP via Cav‐1 rescue experiment. (A) Cav‐1 overexpression could antagonize the inhibitory effect of the high‐cholesterol concentration on BSEP and reverse PKCα phosphorylation; (B) A PKCα inhibitor inhibited PKCα phosphorylation and increased BSEP expression; although the high‐cholesterol concentration still inhibited Cav‐1.

We performed a rescue experiment to further confirm the role of Cav‐1 and PKCα in the regulation of BSEP through cholesterol. We found that, under a high‐cholesterol concentration (pathological condition), Cav‐1 overexpression could antagonize the inhibitory effect of the high‐cholesterol concentration on BSEP and reverse PKCα phosphorylation, indicating that Cav‐1 was indeed regulating BSEP and played an important signalling role in the expression and function of BSEP under cholesterol stimulation (Figure 7A). Subsequently, after addition of a PKCα inhibitor (GO6983), we found that the high‐cholesterol concentration still had an inhibitory effect on Cav‐1, but PKCα phosphorylation was inhibited and the inhibition of BSEP expression was weakened, indicating that the PKCα inhibitor was indeed blocking the regulation of BSEP by Cav‐1 (Figure 7B).

## 4 DISCUSSION

Gallbladder cholesterol stones are one of the most common digestive diseases treated with surgery. A decreased bile salt and phospholipid content in bile along with a relatively supersaturated cholesterol level are two of the fundamental pathologies to cholesterol‐stone formation. BSEP is the most important enzyme regulating bile acid transport. BSEP protein expression levels are regulated by a variety of factors, both at the transcriptional (long‐term regulation) and the post‐transcriptional level (short‐term regulation). Cav‐1 has often been considered a master cell signalling regulator.^15^ In this study, we proposed that Cav‐1 regulated the bile salt export pump on the canalicular membrane of hepatocytes by PKCα‐associated signalling under cholesterol stimulation.

Previous studies^16^, ^17^ on stone disease frequently reported changes in BSEP and Cav‐1 only after stone formation. Here, we also studied an intermediate 2‐week HFC‐fed group during stone formation. We found that a 2‐week HFC diet stimulated Cav‐1 and BSEP expression and decreased PKCα phosphorylation in C57BL/6 mouse liver cells, potentially avoiding gallbladder stone formation. Conversely, long‐term stimulation with HFC diet resulted in decreased Cav‐1 and BSEP expression and increased PKCα phosphorylation, with evident gallbladder stone formation. Ye^18^ found that *MDR2* and *BSEP* mRNA expression levels decreased in the liver of mice with cholesterol gallstones. Pawlikowska^19^ found that patients with a *BSEP* gene deletion or mutation were more likely to develop gallstones and portal hypertension. Kong^20^ reported that BSEP, MRP2 and MDR3 mRNA and protein expression levels were substantially downregulated in the liver tissue of a patient with cholesterol stones, and attributed that downregulation to the cholesterol stone formation. Liu^21^ also confirmed the downregulation of Cav‐1 mRNA and protein levels in the liver tissue of lithogenic mice. These studies were consistent with our results. Furthermore, we added different cholesterol concentrations to HepG2 cells to mimic the different stages of cholesterol stone formation. Under a low‐cholesterol concentration, Cav‐1 and BSEP protein and mRNA expression significantly increased, and PKCα phosphorylation significantly decreased in HepG2 cells. Under a high‐cholesterol concentration, Cav‐1 and BSEP protein and mRNA expression decreased, and PKCα phosphorylation increased in HepG2 cells. This was consistent with the results obtained in our animal model experiments. However, cholesterol stimulation did not significantly affect Hax‐1 expression. A previous study suggested that the motor protein myosin II regulatory light chain (MLC2) could assist in BSEP transport to the apical membrane,^22^ while Hax‐1 could interact with cortactin, promoting BSEP internalization.^23^ Therefore, we speculated that, although cholesterol did not change the Hax‐1 protein content, it could affect Hax‐1 ability to bind to BSEP, thereby affecting BSEP localization in the cell membrane. Under a low‐cholesterol concentration, capacity of BSEP to bind to Hax‐1 was weakened, and BSEP membrane localization as well as transport activity increased; under a high‐cholesterol concentration, ability of BSEP to bind to Hax‐1 was enhanced, Hax‐1 promoted BSEP internalization, BSEP cytoplasmic localization increased, and its transport function was reduced. Therefore, when stimulated by low cholesterol, cells could transport cholesterol through self‐regulation to achieve a balance without requiring any external intervention. Conversely, if stimulated by high cholesterol, promoting the membrane localization of BSEP or enhancing the transport function of BSEP would aid in reducing the cholesterol concentration.

Cav‐1 is the principal structural component of caveolae, which interacts with the cytoskeleton inside the cell and the extracellular matrix outside the cell, and is often considered a master regulator of cellular signalling.^24^ Cav‐1 co‐localizes with cholesterol‐rich membrane domains, and it can induce cholesterol aggregation and membrane bending. Cav‐1 attenuated established atherosclerotic lesions in apoE‐deficient mice, playing an important role in regulating intracellular cholesterol homeostasis,^25^ and also modulated the activity of other proteins involved in the regulation of this process.^26^ Similar to BSEP, a low‐cholesterol concentration could promote Cav‐1 expression, while a high‐cholesterol concentration would inhibit it.

Schwartz^27^ found that the PKCα content in epithelial cells was considerably increased after feeding mice with a high‐cholesterol diet. In a cholesterol‐fed prairie dog study, PKCα mRNA and protein expression substantially increased during gallstone formation, and the Na^+^/H^+^ exchange capacity decreased.^28^ Furthermore, PKCα could be a sensor of cell membrane pressure.^29^ Contrary to BSEP and Cav‐1, a low‐cholesterol concentration could inhibit PKCα phosphorylation, while a high‐cholesterol concentration could promote it.

Subsequently, we found that Cav‐1 overexpression reduced the phosphorylation of PKCα, increased BSEP protein expression, enhanced BSEP membrane localization and weakened BSEP's capacity to bind to Hax‐1. Previous literature showed that C57BL/6 mice infected with recombinant human Cav‐1 and Cav‐2 adenoviruses presented an increased bile flux and secretion of all bile lipids.^30^ After 2 weeks of intravenous injection of the antenapedia‐Cav‐1 peptide in hypercholesterolemic rabbits, Cav‐1 expression increased by 15%, improving the dyslipidemia and inhibiting the cyclophilin A mediation, leading to the production of reactive oxygen species and improvement in mitochondrial function.^31^ Recently, Fernandes^8^ used cyclic, square wave and differential pulse voltammetry on glassy carbon electrodes for the first time to study the difference in Cav‐1 peak oxidation current under different cholesterol conditions, proving that Cav‐1 was directly related to cholesterol transport. The above studies and our results suggested that Cav‐1 played a positive role in cholesterol efflux. We further tried to construct a Cav‐1 knockdown cell line, but after knockdown of Cav‐1, cell viability decreased and all cells died in a short period of time. After knockdown of Cav‐1 in mesenchymal stem cells, the caveolae content, signal transmission and biological activity of these cells were reduced.^32^ A mouse model of hindlimb ischemia in male endothelial‐specific Cav‐1 knockout mice showed that the degree of microvascular sprouting was substantially reduced and blood flow recovery was considerably impaired in these mice.^33^ These studies and our results suggested that stable expression of Cav‐1 might be a key factor in cell signalling and cell survival.

To further confirm the role of Cav‐1 and PKCα in the regulation of BSEP by cholesterol, we performed a rescue experiment: under a high‐cholesterol concentration (pathological condition), Cav‐1 overexpression could antagonize the inhibitory effect of the high‐cholesterol concentration on BSEP and reverse PKCα phosphorylation. Subsequently, after adding a PKCα inhibitor, we found that the high‐cholesterol concentration still had an inhibitory effect on Cav‐1, but PKCα phosphorylation was inhibited, and the inhibition of BSEP expression was weakened, indicating that PKCα had indeed an important role in Cav‐1 regulation of BSEP expression and function on the canalicular membrane in hepatocytes under cholesterol stimulation. This article has some limitations, which should be noted. First, the phosphorylation of Cav‐1 and Hax‐1 was not studied. Second, the specific binding mechanism of BSEP and Hax‐1 was not thoroughly explored. Lastly, due to sampling of human specimens, no relevant protein analysis was performed.

## 5 CONCLUSION

**FIGURE 8:**
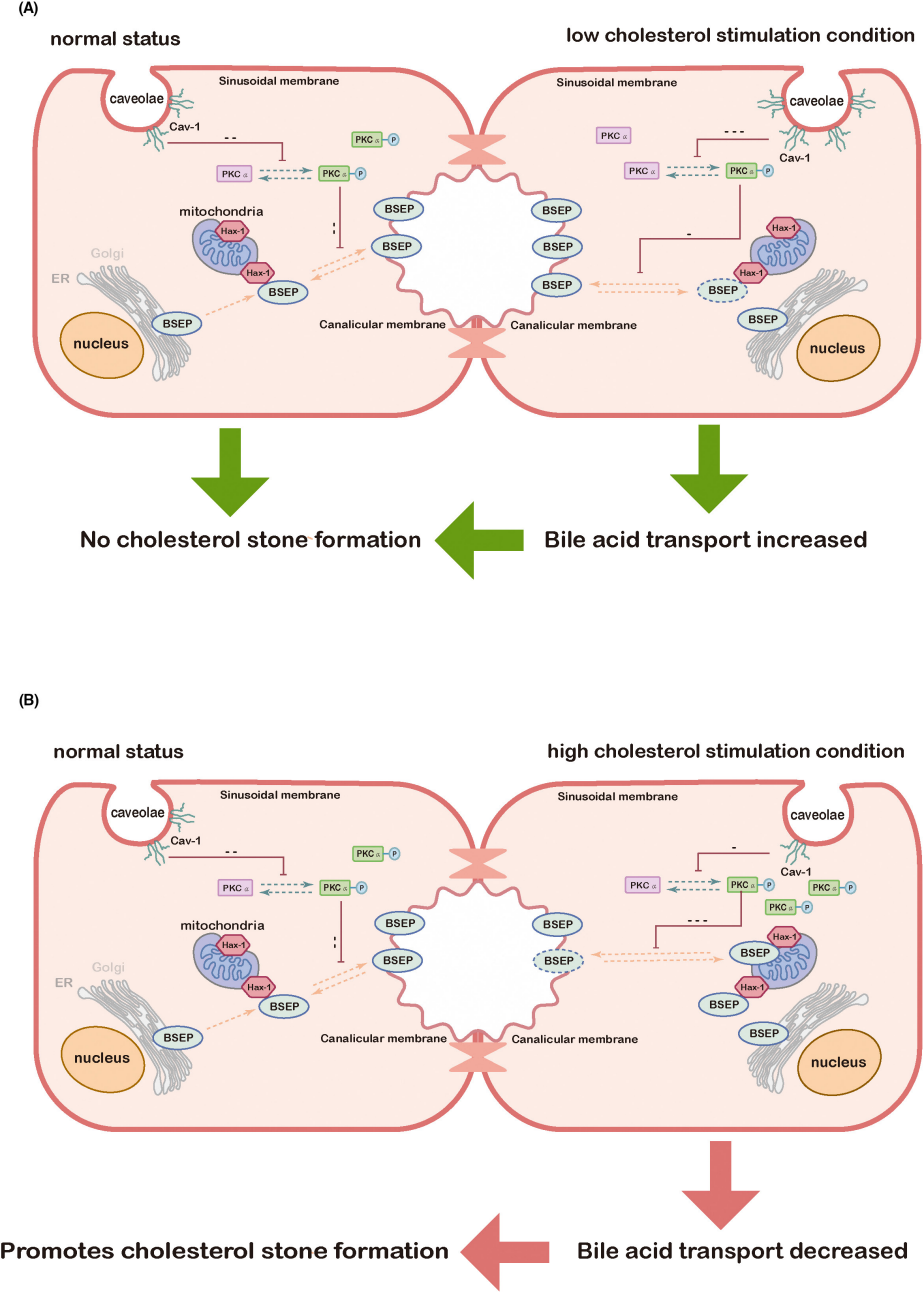
Mechanism of regulation of BSEP expression and function in HepG2 cells through cholesterol via Cav‐1. (A) Under a low‐cholesterol concentration, Cav‐1 expression was enhanced, PKCα phosphorylation was inhibited, the binding of BSEP to Hax‐1 was attenuated, and BSEP membrane expression and transport function were enhanced; (B) Under a high‐cholesterol concentration, Cav‐1 expression was inhibited, PKCα phosphorylation was enhanced, the binding of BSEP to Hax‐1 was enhanced, BSEP internalization was promoted, and BSEP membrane expression and transport function were weakened, leading to the promoted formation of cholesterol stones.

Altogether, our findings suggested that Cav‐1 regulated the bile salt export pump on the canalicular membrane of hepatocytes via PKCα‐associated signalling under cholesterol stimulation. Under a low‐cholesterol concentration, Cav‐1 expression was enhanced, PKCα phosphorylation was inhibited, BSEP's ability to bind to Hax‐1 was reduced and BSEP expression and transport function were enhanced. Under a high‐cholesterol concentration, Cav‐1 expression was inhibited, PKCα phosphorylation was enhanced, BSEP's ability to bind to Hax‐1 was enhanced, BSEP internalization was promoted and BSEP membrane expression and transport function were attenuated (Figure 8).

## AUTHOR CONTRIBUTIONS

**Liwei Pang:** Data curation (lead); formal analysis (equal). **Meiying Cui:** Writing – review and editing (equal). **Shuodong Wu:** Writing – review and editing (equal). **Jing Kong:** Writing – review and editing (equal).

## FUNDING INFORMATION

This study was funded by the National Nature Science of China (81670580) and 345 Talent Project and Shenyang Science and Technology Innovation Talent Support Program for Youth and Midlife (RC200121).

## CONFLICT OF INTEREST STATEMENT

The authors declare no conflict of interest.

## Supporting information


Appendix S1

## Data Availability

Data available on request.
