# Supplementary material for: Cav‐1 regulates the bile salt export pump on the canalicular membrane of hepatocytes by PKCα‐associated signalling under cholesterol stimulation
Source: J Cell Mol Med. 2024 Jan 1;28(3):e18110. doi: 10.1111/jcmm.18110 (PMC10844719; doi:10.1111/jcmm.18110)
Supplement: Supplementary file 1 — Appendix S1 [file JCMM-28-e18110-s001.doc]

（1）Cells were collected after 48 hours of cholesterol loading with different concentrations

（2）Determination of protein concentration by BCA method

（3）Preparation of taurocholic acid and orthovanadate solution: prepare 10mM TC solution and dilute it to 1mM with reagent I in ATPase activity detection kit. Weigh 100mg of orthovanadate (which can inhibit BSEP) powder and dissolve it in 10mL of reagent I to prepare 25mM orthovanadate solution. Spare after filtering.

（4）Enzymatic reaction: the reaction is carried out in EP tube, and two groups of control group and inhibition group are set, and the control group is added with 5μl Reagent 1, inhibition group added 5μl 25mM orthovanadate solution. The two groups add reagent 1 and 1mM substrate (as shown in the table below) according to the substrate concentration set in each tube, and then add 100μl sample, preheat the mixture to 37℃ for 5 minutes. Then add 40μl Reagent II, 20μl Reagent III and 20μl Reagent IV is fully mixed to start the reaction. Each tube is accurately bathed at 37℃ for 20 minutes, and 25μl Reagent V was mixed evenly to terminate the reaction. Centrifuge 4000g of each tube at room temperature for 10 minutes, and take the supernatant for test.

| Substrate concentration（μM） | 0 | 10 | 20 | 40 | 80 |
| --- | --- | --- | --- | --- | --- |
| Reagent I（μl） | 40 | 37.5 | 35 | 30 | 20 |
| 1mM substrate（μl） | 0 | 2.5 | 5 | 10 | 20 |

（5）Preparation of phosphorus determination reagent: mix ddH2O: Reagent VI: Reagent VII: Reagent VIII to prepare phosphorus determination reagent in the ratio of 2:1:1

（6）Phosphorus determination: blank tube and standard tube are set with 2 multiple holes, and control tube and inhibition tube are set with 3 multiple holes. Add 20μl in each hole according to the following table Standard phosphorus application solution (0.5μmol/ml), supernatant or distilled water, then add 200μl Phosphorus determination reagent, placed at 37℃ for 30-60 minutes.。

|  | Blank | Standard | Contrast | Suppression |
| --- | --- | --- | --- | --- |
| Standard phosphorus application solution（μl） |  | 20 |  |  |
| Supernatant（μl） |  |  | 20 | 20 |
| Distilled water（μl） | 20 |  |  |  |

（7）Absorbance measurement: preheat the microplate for more than 30 minutes, adjust the wavelength to 660 nm, shake the plate for 10 seconds after the distilled water is zeroed, and then measure and record the OD value of each hole.

（8）ATPase activity calculation: It is specified that the amount of 1 nmol inorganic phosphorus produced by the decomposition of ATP by BSEP in each milligram of protein per minute is one enzyme activity unit (U).

BSEP transport capacity sensitive to orthovanadate (U/ug prot)=C standard tube × (A control tube - A suppression tube) ÷ (A standard tube - A blank tube) × V total ÷ (Cpr × V sample) ÷ T=62.5 × (A control tube - A suppression tube) ÷ (A standard tube - A blank tube) ÷ Cpr

Remarks: C standard tube: standard tube concentration, 0.5 μmol/mL； Total V: total volume of enzymatic reaction, 0.25 mL; V sample: added sample volume, 0.1 mL; T: Reaction time, 20 minutes; Cpr: sample protein concentration, mg/mL.
